# Effects of a Randomized Home-Based Quality of Movement Protocol on Function, Posture and Strength in Outpatients with Obesity

**DOI:** 10.3390/healthcare9111451

**Published:** 2021-10-27

**Authors:** Luca Cavaggioni, Luisa Gilardini, Gabriella Redaelli, Marina Croci, Paolo Capodaglio, Michele Gobbi, Simona Bertoli

**Affiliations:** 1 Department of Biomedical Sciences for Health, Università Degli Studi di Milano, 20129 Milan, Italy; 2 Obesity Unit and Laboratory of Nutrition and Obesity Research, Department of Endocrine and Metabolic Diseases, Istituto Auxologico Italiano IRCCS, 20145 Milan, Italy; l.gilardini@auxologico.it (L.G.); g.redaelli@auxologico.it (G.R.); m.croci@auxologico.it (M.C.); simona.bertoli@unimi.it (S.B.); 3 Rehabilitation Unit and Research Laboratory in Biomechanics and Rehabilitation, Istituto Auxologico Italiano IRCCS, San Giuseppe Hospital, 28824 Verbania, Italy; p.capodaglio@auxologico.it (P.C.); m.gobbi@auxologico.it (M.G.); 4 Department of Surgical Sciences, Physical Medicine and Rehabilitation, University of Turin, 10124 Turin, Italy; 5 International Center for the Assessment of Nutritional Status (ICANS), Department of Food, Environmental and Nutritional Sciences (DeFENS), University of Milan, 20133 Milan, Italy

**Keywords:** obesity treatment, movement quality, physical fitness

## Abstract

The aim of this study was to determine the effects of two different home-based training interventions on functional parameters and body composition in obese patients. Sixty-four obese patients were recruited at the IRCCS Istituto Auxologico Italiano and randomly assigned into a movement quality group (MQ) and a conventional training group (CT). In the MQ, the training protocol combined various stimuli based on whole-body movement patterns, mobility, motor control and diaphragmatic breathing. The CT included traditional bodyweight resistance-training exercises. All patients were tested for movement efficiency (Functional Movement Screen, FMS), postural control (Modified Balance Error Scoring System, M-BESS), breathing pattern (Total Faulty Breathing Scale, TFBS), muscular strength (Handgrip Strength Test, HST and Five Repetition Sit to Stand, FRSTS) and body composition (Waist Circumference, WC, Body Mass Index, BMI, Body fat mass percentage, Fat Mass) before and after a 6-week period of training. Significant interactions and main effects of time (*p* < 0.0001) were found in MQ compared to CT in the FMS, M-BESS and TFBS parameters, while muscular strength (HST, FRSTS) and body composition parameters improved similarly in both groups with a main effect of time (*p* < 0.05). These findings suggest that a 6-week movement quality training is effective in ameliorating postural control and movement efficiency with similar improvements in muscular strength and body composition compared with a mere traditional home-based training. Fitness coaches and practitioners might consider the MQ intervention as a valuable alternative to conventional training when treating obesity.

## 1. Introduction

Being physically fit may play a valuable role in the development of wellbeing or injury prevention [1,2,3] and within the parameters related to the decrease of physical fitness, obesity is one of them [4]. In this context, ample evidence demonstrates that obesity is associated with an augmented cardio-metabolic risk [5], reduced postural control [6], dynamic balance [7,8], functional movement performance [9,10], strength-related variables [11] and breathing-pattern efficiency [12]. In light of this, obese patients might have several compensatory strategies to cope with reduced motor control because, as body weight increases, higher muscular strength is required to stabilize the center of pressure within the base of support [13,14]. In particular, it was observed that excessive body weight diminishes limbs and trunk strength, leading to impaired muscle function [15].

Notably, Capodaglio et al. (2009) suggested that an obesity-related rehabilitation program should consider components such as proprioception, muscular isometric strength, cardiorespiratory conditioning and body-image exercises [6] respecting a multidisciplinary approach [16]. International guidelines of the American College of Sports Medicine and World Health Organization state that to improve fitness levels at least 150 min a week of physical activity at moderate intensity, combined with resistance training composed of 8–12 repetitions with an intensity of 60–70%1RM for each muscle group, are recommended [17,18,19]. However, the widely accepted aforementioned guidelines mainly focus on quantity of exercise with little consideration regarding the quality of motor execution (i.e., “how” an individual performs each exercise).

As for the effectiveness of quality movement training on physical fitness and postural control, the available literature is scarce and there is a prevalence of studies conducted on healthy, normal-weight individuals [20,21].

In this regard, Frost et al. (2015) enrolled healthy adults assigned into three groups: a “movement-guided”, a conventional fitness and a control group. After 12 weeks of training, the first 2 groups showed significant improvements in fitness parameters (aerobic capacity, muscle strength, flexibility), but only the “movement-guided” group showed an amelioration in the biomechanical evaluation (kinematic analysis of lumbar spine during whole-body tasks), meaning a better motor control [21]. A further study by Bennett et al. (2019) aimed to compare the potential effects of a quality movement training regimen on “fitness” (countermovement jump, broad jump, single leg broad jump, 3RM deadlift, 3RM bench press and 3RM row) and “functional” variables (Functional Movement Screen and MovementSCREEN). Both intervention groups (“quality movement” and “traditional fitness”) demonstrated significant improvements in fitness parameters overtime with no between-group differences. On the contrary, solely the “quality movement” group showed significant improvements in functional performance measurements [20]. Overall, these experimental studies provide additional evidence regarding the positive effects of a quality movement training program on fitness levels and postural control in normal-weight individuals.

To the best of our knowledge, no previous studies evaluated the impact of a quality movement training regimen on patients with obesity.

Therefore, the aim of this study was to evaluate whether a quality movement exercise protocol would be more effective than a traditional resistance training in enhancing postural control and fitness parameters in patients with obesity.

## 2. Materials and Methods

### 2.1. Participants

The study sample included 64 Caucasian patients with obesity recruited among the patients referred to the IRCCS Istituto Auxologico Italiano for a weight management intervention. Exclusion criteria were patients aged more than 70 years, patients with knee pain (Visual Analogue Scale > 7 a.u.), history of hip or knee replacement, severe hip or knee ostheoarthrosis, cardiac or neurological and any other medical conditions contraindicating physical activity.

### 2.2. Multidisciplinary Lifestyle Program

The lifestyle program consisted of 1 weekly 3-h session including a medical examination, nutritional education, peer group psychological support and individual consultancies regarding physical activity for a comprehensive duration of 3 months. A self-monitored diary including food consumption and daily physical activity were used as a tool for education and reinforcement. Diet based on a caloric restriction (1200–2000 kcal/day, 21% proteins, 53% carbohydrates, 26% lipids) was prescribed by a dietitian to each participant.

### 2.3. Procedures

Within the lifestyle program, patients were randomly assigned to two physical activity intervention groups as determined by a random number generator on a computer performing a block randomization design: Movement-Quality group [MQ, *n* = 32] and Conventional Training group [CT, *n* = 32]. At the end of the study, we checked that no errors had been made in allocation. The investigator (LC) generated the random allocation sequence, two other investigators (GR and MC) enrolled participants, and one investigator (LG) assigned participants to interventions. For each subject, an individual home-based training program was prescribed by a certified sport scientist for 6 consecutive weeks (total training sessions: 18). During this time, patients were asked not to perform other physical exercise aside from that specified for the purposes of this study. Before and after the 6-week intervention, functional parameters regarding movement efficiency, breathing pattern and muscular strength were performed. Finally, at baseline and at the end of the multidisciplinary lifestyle program measurements on body composition parameters were conducted.

#### Functional Parameters

The functional movement performance was assessed using the Functional Movement Screen (FMS). This testing procedure was developed by Cook and Burton [22,23] and consists of performing seven basic locomotor patterns. According to Molina-Garcia (2019), to overcome execution difficulties presented by patients with obesity, only four patterns were considered (deep squat, hurdle step, shoulder mobility and active straight leg raise). Movement efficiency is graded on a scale from 0 to 3 points based on how a task is executed. Each single score was summed to provide a final composite score. Administration of the FMS was provided by a certified instructor. This testing procedure has a moderate to high inter-rater reliability (Kappa coefficient = 0.64, 0.57, 0.76, and 0.79) [9].

Static postural control was measured using the modified version of the Balance Error Scoring System (M-BESS) [24]. M-BESS requires participants to stand in an upright position with hands on hips, eyes closed on a firm surface for 20 s under three conditions: double stance, tandem and single stance. During each trial, the examiner counts the number of errors that the participant performs according to the following criteria: moving hands off hips, opening eyes, stepping, stumbling, or falling, shifting hips more than 30 degrees of flexion or abduction, lifting the heel or the forefoot, remaining out of the proper testing position more than 5 s. The total number of errors were summed to produce M-BESS score. M-BESS shows a high inter-rater reliability (ICC = 0.88) [24].

The faulty breathing was assessed using the Total Faulty Breathing Scale (TFBS) [25]. The examiner observed the patient’s breathing pattern in terms of abdominal contribution, lower rib expansion or upper ribs–clavicle motion. The scoring was based on three main criteria: absence of outward lateral rib motion (score “1”), lifting of the clavicle (score “2”) and paradoxical breathing (score “3”) in both relaxed and deep breathing. This testing procedure presents a high inter-rater (Kappa score 1) and intra-rater reliability (Kappa score 0.780) [25].

To detect muscular strength, the Handgrip Strength Test (HST) and the Five Repetition Sit-To-Stand test (FRSTS) were performed. The HST was conducted with a hydraulic dynamometer Jamar (J. A. Preston Corporation, Clifton, NJ, USA) in respect to the standardized testing procedures [26] whose validity and reliability (r = 0.81) were previously established [27]. The patient was seated on a chair without armrests with the elbow flexed at 90° exerting the maximum isometric grip. Three measurements for each hand are performed with a 45-s rest in between. The mean value was calculated for the subsequent analysis. In the FRSTS test, the patient should rise from the chair as quickly as possible for five times consecutively with his/her arms crossed over the chest [28,29]. Three measurements using a manual stopwatch were conducted with 90 s of rest. This testing procedure exhibits a high intra-rater reliability coefficient (ICC = 0.81) [29].

Clinical and body Compositional measures, Body Mass Index (BMI), was derived from anthropometric measurements calculated as weight (kilograms) divided by square of height (meters squared). Body weight was measured to the nearest 0.1 Kg with a calibrated weight scale (SECA^®^ 877, Hamburg, Germany) and body height was recorded to the nearest 0.1 cm with a stadiometer (SECA^®^ 240, Hamburg, Germany).

Waist Circumference (WC) was measured at the level of the umbilicus with the patient in quite standing position during the expiration phase.

Body fat mass percentage (FM%) was assessed using bioelectric impedance analysis (BIA 101-RJL Systems Akern srl, Firenze, Italy) by a trained nurse during which the patient was positioned in a supine position abducting his/her lower limbs of 45° with respect to the midline of the body. It was requested that the patient fast overnight and not practice of physical activity in the previous 12 h. The bioelectrical impedance assay was performed in the morning after resting for 20 min, in a well-ventilated room with constant temperature.

### 2.4. Training Intervention

The two home-based training protocols consisted of a 6-week exercise program designed to improve general fitness levels, but differed with regard to the type, order of presentation of the exercises and movement pattern performed.

The MQ protocol followed the approach reported in the study of Cavaggioni et al. (2019) emphasizing mobility, stability and neuromuscular training [30]. In detail, it was based on self-awareness about the movement execution while performing multi-joint strength exercises [31], diaphragmatic breathing [32,33] and corrective postures to emphasize motor control [22,23]. Conversely, the CT program was chosen from a variety of traditional strength exercises with the primary objective to increase fitness levels using bodyweight and elastic-bend exercises following the recommendations proposed by the American College of Sports Medicine [19].

A detailed description of the overall training interventions is shown in Table 1. Both protocols were administered in a circuit-training modality (time of work per set 30 s, time of rest between each exercise 15 s, time of rest between sets 2 min, total volume 3 sets) for a duration of 45 min with a frequency of 3 days per week, respectively.

The training adherence was monitored by weekly individual consultancy with a certified sport scientist. It was based on the proper exercise technique correction and the verification as concern the compilation of a daily diary about home-training (training date, duration of the entire session and rating of perceived exertion RPE, CR-10 scale).

### 2.5. Ethical Considerations

The study was approved by the Ethics Review Committee of the IRCCS Istituto Auxologico Italiano, Italy. All subjects provided written, voluntary, informed consent before participating and the experiment was conducted in according to the Declaration of Helsinki. This randomized controlled trial was compliant to all TIDieR checklist [34].

### 2.6. Statistical Analysis

All statistical analyses were performed using the Statistical Package for Social Sciences IBM™ SPSS™ Statistics (version 21.0, IBM Corp., Somers, Chicago, IL, USA).

All data are presented as mean ± standard deviation (SD). The dataset was tested for normal distribution using Shapiro–Wilk’s normality test. Sample-size calculation was established on power analysis (α = 0.05, β = 0.72, effect size = 0.77) on the basis of a previous study examining the effects of a movement quality training on normal-weight individuals [20].

A mixed two-way repeated measures analysis of variance (ANOVA) was used to determine the interactions between the two independent variables (pre/post treatment) and group (MQ and CT group) on both functional and obesity parameters. Partial eta squared (Part η2) effect size was used to estimate the magnitude of the difference within each group and a threshold for small, moderate and large effects were defined as 0.01, 0.06 and 0.14, respectively [35]. To examine differences in each single task of the FMS parameter, due to the rank-order nature of the data, a Mann–Whitney U test was used. To detect if changes are clinically meaningful, the Minimal Clinical Important Difference (MCID) value was obtained by multiplying the standard deviation of the baseline scores by 0.2 (i.e., MCID = 0.2 × SD) [36]. An alpha value of *p* < 0.05 was set as criterion level of significance.

## 3. Results

A total of 100 patients were assessed for eligibility and were allocated either to the MQ group or to the CT intervention. Twenty-five of the eligible patients (25%) did not meet the inclusion criteria and were excluded. Eleven eligible participants dropped out (15%), and the final sample comprised sixty-four patients who were used for statistical analysis. No adverse situations were reported during the study period.

**Figure 1:**
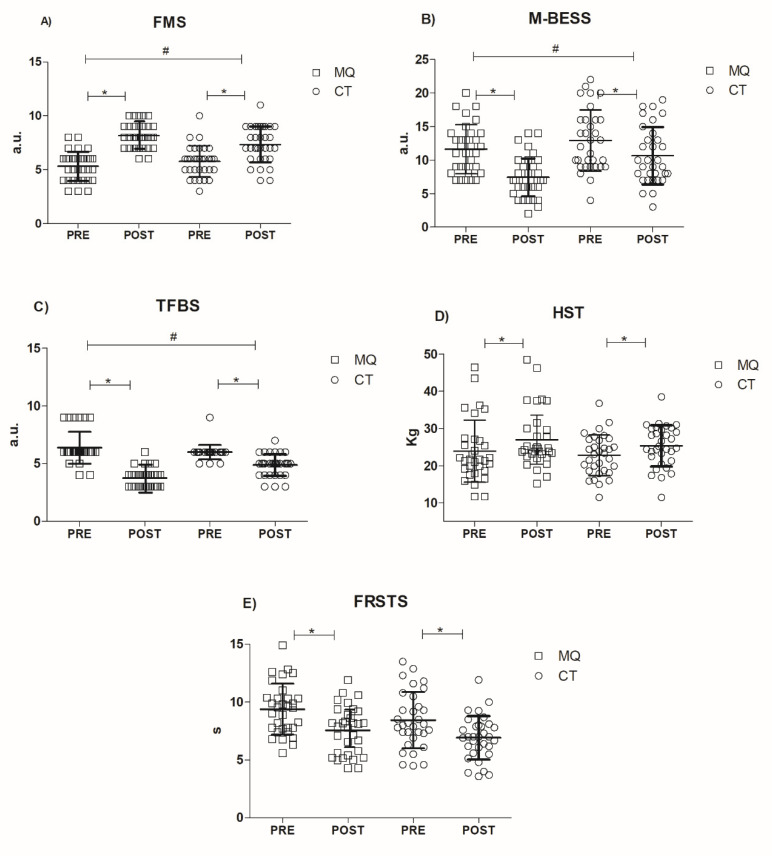
Functional parameters (“(**A**)” FMS Composite Score, “(**B**)” M-BESS, “(**C**)” TFBS, “(**D**)” HST, “(**E**)” FRSTS). * Significant main effect of time before and after testing (*p* < 0.05). # Significant interaction (*p* < 0.0001).

**Figure 2:**
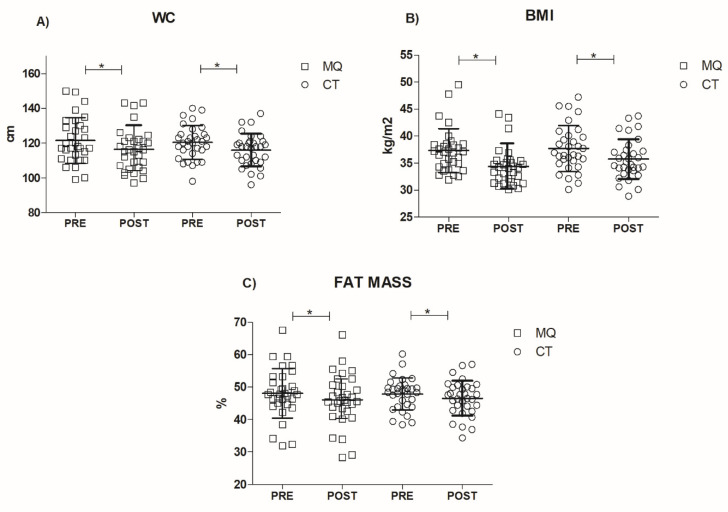
Clinical and nutritional status parameters (“(**A**)” Waist Circumference, “(**B**)” Body Mass Index, “(**C**)” fat mass percentage). * Significant main effect of time before and after testing (*p* < 0.05).

At the baseline, both groups presented non-significant differences (*p* > 0.05) in functional parameters and in mean age, sex, body mass and FM % (Table 2). The effect of training intervention programs on functional, clinical and body compositional parameters are presented in Figure 1 and Figure 2.

As concerns the FMS Composite Score a significant interaction was found (F1,26 = 259.93, *p* = 0.000016, part η2 = 0.80) (Figure 1A). In addition, a significant main effect of time (pre-to-post) (F1,26 = 259.9, *p* < 0.0001, part η2 = 0.80) was detected, but no main effect of group (F1,26 = 0.276, *p* = 0.602) was found. It should be noted that for both groups the effect size (MQ d = 2.2; CT d = 1.1) was superior compared to the MCID value of 0.3. As regards each FMS single task, it is possible to appreciate a significant difference in the Hurdle Step (*p* value = 0.0036) and in the Active Straight Leg Raise tasks (*p* value = 0,0003) in favor of MQ compared to the CT group.

In M-BESS, the total score showed a significant interaction (F1,26 =17.05, *p* = 0.000110) (Figure 1B) with a significant main effect of time (F1,26 = 196.41, *p* < 0.0001, part η2 = 0.76), and main effect of group (F1,26 = 5.58, *p* = 0.021). It should be pointed out that the MQ group effect size (d = 1.2) was clinically meaningful compared to the related MCID (0.7).

The Total Faulty Breathing Scale composite score revealed a significant interaction (F1,26 = 201.30, *p* = 0.00002, part η2 = 0.26) with a main effect of time (F1,26 = 201.37, *p* < 0.0001, part η2 = 0.76) (Figure 1C), without a significant main effect of group (F1,26 = 0.08, *p* = 0.77). Both groups highlighted that their effect size (MQ d = 2.3; CT d = 0.8) were superior compared to the MCID value of 0.3.

Finally, both HST and FRSTS did not reveal any significant interactions (F1,26 = 2.01, *p* = 0.161; F1,26 = 0.76, *p* = 0.385) (Figure 1D,E). Conversely, only a significant main effect of time in HST (F1,26 = 212.7, *p* < 0.0001, part η2 = 0.70) and in FRSTS (F1,26 = 98.05, *p* < 0.0001, part η2 = 0.61) were observed, with no main effect of group (*p* > 0.05).

Lastly, Body Mass Index, Waist Circumference, and fat mass percentage did not highlight any significant interaction or main effect of group (*p* > 0.05), revealing only a significant main effect for time (“WC” F1,26 = 156.78, *p* < 0.0001, part η2 = 0.72; “BMI” F1,26 = 128.73, *p* < 0.0001, part η2 = 0.67; “FM%” F1,26 = 14.78, *p* < 0.0001, part η2 = 0.19) (Figure 2A–C).

## 4. Discussion

The present study aimed to investigate the effectiveness in obese individuals of a 6-week home-based on movement quality training compared with a conventional training stimuli on functional movement performance, breathing pattern, muscular strength and body composition. The main findings were that the MQ group performed significantly better than the CT in the FMS, M-BESS and TFBS tests after the training period. Interestingly, MQ and CT responded similarly to training in strength-related variables and anthropometric parameters.

As concern functional parameters, we observed in MQ a significant improvement FMSTM composite score exhibiting a better movement efficiency compared to CT. Specifically, this amelioration may have been provided by the nature of the training program itself. In fact, the MQ training protocol was designed on the interplay that is a key factor to enhance functional movement competency [37]. According to this, ample evidence demonstrates a significant association between functional performance and the Body Mass Index in both adults and children [9,10]. Nevertheless, despite the excess body weight that affects mobility and stability [38], a specific training intervention that emphasizes self-awareness may be a suitable approach in treating obesity [39]. Our results are in line with those depicted by Bennett et al. (2019), but it should be mentioned that our MQ group recorded a remarkable increase of 2.8 points (53%) compared to only 1.7 (13%) as observed in Bennett’s movement quality training group [20].

Regarding the M-BESS results, our data are in line with the findings reported in normative studies [24] observing, at baseline, a poor postural control. After the intervention period in our MQ group there was a significant increase in body balance exhibiting a broadly normal score in respect to the CT group whose score remained poor. The concomitant reduction in body weight and the increased synergy in postural control systems (i.e., visual, vestibular, somatosensory) promoted by MQ exercises may have been beneficial in the movement quality training group over time. An excessive body weight may alter the center of mass displacement [13] with a concomitant reduction in medio-lateral stability [6] imposing new biomechanical adaptations to maintain the balance [7]. Our results led us to speculate about the importance of including a component of balance exercises during obesity-rehabilitation interventions to cope with the negative consequences of an excess of body weight on postural control.

For what concerns the breathing-pattern efficiency, the TFBS results recorded in the MQ group showed a significant change pre-to-post condition, passing from a moderate to a mild breathing pattern dysfunction. On the contrary, the CT group denoted a moderate dysfunction throughout the entire intervention period. In light of this, many lines of evidence support the notion that obesity may have a negative impact on ribcage motion due to fat accumulation in the thoraco-abdominal compartments [40,41]. Presumably, both the reduction in body weight and the specific nature of MQ exercises could have displayed a valuable role in ameliorating the rib-cage expansion.

Moreover, muscular strength showed a significant amelioration during the intervention period in both groups. In light of this, Hills et al. (2002) provide direct evidence that obese individuals who showed reduced strength values might have a reduced motor function that could limit everyday-life activities [42]. In our sample, the HST strength values were quite lower than normative data reported by Massy-Westropp et al. [43]. In any case, both interventions were designed to improve neuromuscular efficiency and, from a speculative point of view, it should be pointed out that the peculiarity of the MQ program was to reinforce whole-body movement patterns in a more functional way. This modality may have gained similar strength ameliorations as the conventional training program. As regards lower-body strength, our baseline FRSTS values were similar to those reported by Hergenroeder et al. (2011) [44]. The reduction in chair rising time obtained in the MQ and CT groups after the intervention period may reflect an augmented lower-body functional capacity. In fact, an increased lower-body strength, provided by both training regimens, might have been fundamental in ameliorating the sit-to-stand performance. Nevertheless, the results reported in the current study are consistent with those of previous studies on normal-weight individuals displaying improvements in limb strength following a training program focused on quality of movement [20,21].

Regarding clinical and body compositional parameters, both groups demonstrated significant pre-to-post changes in BMI, Waist Circumference and fat mass percentage with no differences between groups across any of the measures. A study by Kraemer et al. (1999) confirmed the effectiveness regarding the combination of physical exercise (i.e., resistance training) with a controlled dietary regimen in improving weight loss [45]. It is worth noting that the exercise modality particularly suited for obese individuals (i.e., circuit training) also might have influenced the final outcomes [46]. From a speculative point of view, observing body compositional results that had a significant increase over time in both groups similarly, led us to hypothesize that the body composition amelioration should be acknowledged mostly by the nutrition treatment and partially derived from the exercise intervention.

In summary, it should be highlighted that an efficient obesity treatment should be conducted in a multidisciplinary way [16], in which a movement-quality oriented physical activity, an individualized nutritional plan and psychologic support are key factors that would accompany the patient during the entire rehabilitation program. Obviously, we do not state that our home-based MQ protocol should replace a traditional gym program, but fitness coaches and practitioners should consider it as a complementary solution to conventional training when managing patients with obesity.

The present study presents three main limitations that should be acknowledged. First, the relatively small sample size and the lack of a control group who did not performed any physical activity intervention makes it difficult to generalize our results to common people. Second, the heterogeneity in participants’ training level and gender differences may have contributed to explain any strength or balance adaptations that occurred in response to training interventions. Third, the exercise equipment used during home training (i.e., water bottles, elastic bands, dumbbells), compared with the equipment used in a gym setting, could have modified the intensity and volume of both resistance training programs and might have influenced our final outcomes. Finally, the nutritional and the psychological support provided within the intervention program may have interacted with the final results. Therefore, a lack of measure of these parameters should be addressed as a limitation.

Future studies should compare the efficacy of this training mode considering different variables such as diet and participants’ age, as well as investigate the effects of a home-based training in the absence of supervision.

## 5. Conclusions

The current study provides evidence that obese individuals who perform a 6-week movement quality home-based training could obtain greater improvements in postural control and breathing-pattern efficiency with similar ameliorations in muscle strength and body composition, compared to conventional training. Additionally, when treating obesity, our findings provide theoretical support for the integration of a movement quality training along with the patient weekly training routine, having the advantage to emphasize motor control and body awareness while practicing physical activity.

## Figures and Tables

**Table 1:** Training content of the 6-week intervention performed by MQ and CT groups.

Weeks	MQ	CT
Week 1 to 2	Wall slide breathing with arms, rib-grab thoracic spine rotation from split stance, hip-airplane both feet on the ground, unilateral horizontal push with band in split squat position, goblet squat with dumbbell, side plank rotation with hands on the wall, unilateral horizontal pull with band in split squat position, in place standing cross-marching, pallof press from standing	Shoulder mobility circumduction, forward spine flexion from standing, quadriceps stretch, wall push-up, abdominal crunch, wall squat exercise, alternated biceps curl with dumbbells, abdominal oblique crunch, alternating forward lunge
Week 3 to 4	Alternated wall slide breathing with arms, thoracic spine rotation with arm reach from split stance, hip-airplane with one foot raised, unilateral horizontal push with band with forward lunge, goblet squat to overhead press with dumbbell, tortional buttress with hands on table/wall, unilateral horizontal pull with band with backward lunge, in place standing cross-marching, stability lift with band in standing position	Shoulder flexion-extension mobility, forward spine flexion from standing with lower-legs abducted, quadriceps stretch, table push-up, abdominal crunch with slow eccentric phase, squat with dumbbells, symmetric biceps curl with dumbbells, abdominal oblique crunch, continuous forward lunge
Week 5 to 6	Hip hinge with dowel, thoracic spine rotation with arm sweep from split stance, diaphragmatic breath with arms raised overhead, unilateral horizontal push with in monopodalic stance, squat with spine rotation and overhead press stability chop with band in standing position, unilateral horizontal pull in monopodalic stance, curl to press with dumbbells, stability lift with band in standing position	Shoulder adduction-abduction mobility, lateral spine flexion from standing with lower-legs abducted, quadriceps stretch, wall push-up with band around scapula, abdominal crunch with slow eccentric phase, squat with band under feet, symmetric biceps curl with band, abdominal oblique crunch, forward lunge with band under anterior foot

**Table 2:** Baseline characteristics highlighted by MQ and CT groups.

Characteristics	MQ	CT
Age (years)	50.5 ± 10.4	50.4 ± 10.7
Sex (Male/Female)	6/26	5/27
Weight (kg)	100.6 ± 12.3	99.0 ± 14.9
BMI (kg/m^2^)	37.3 ± 4.0	37.7 ± 4.2
Fat mass (%)	37.3 ± 4.0	37.3 ± 4.0
IPAQ (METS)	552 ± 810	566 ± 1094
Functional Movement Screen^TM^ (a.u.)	5.3 ± 1.4	5.8 ± 1.4
Modified Balance Error Scoring System (a.u.)	11.6 ± 3.7	12.9 ± 4.6
Total Faulty Breathing Scale (a.u.)	6.4 ± 1.4	5.6 ± 1.6
Handgrip Strength Test (kg)	23.9 ± 8.3	22.8 ± 5.5
Five repetition sit-to-stand	9.4 ± 2.2	8.4 ± 2.4

## Data Availability

The data are not publicly available due to privacy reasons.
